# Eight years of IMRT quality assurance with ionization chambers and film dosimetry: experience of the montpellier comprehensive cancer center

**DOI:** 10.1186/1748-717X-6-85

**Published:** 2011-07-20

**Authors:** Pascal Fenoglietto, Benoit Laliberté, Norbert Aillères, Olivier Riou, Jean-Bernard Dubois, David Azria

**Affiliations:** 1 Département de Cancérologie Radiothérapie et de Radiophysique, CRLC Val d'Aurelle-Paul Lamarque, Montpellier, France; 2 Département de Radio-Oncologie, Hôpital Maisonneuve-Rosemont, Montréal, Canada

## Abstract

**Background:**

To present the results of quality assurance (QA) in IMRT of film dosimetry and ionization chambers measurements with an eight year follow-up.

**Methods:**

All treatment plans were validated under the linear accelerator by absolute and relative measures obtained with ionization chambers (IC) and with XomatV and EDR2 films (Kodak).

**Results:**

The average difference between IC measured and computed dose at isocenter with the gantry angle of 0° was 0.07 ± 1.22% (average ± 1 SD) for 2316 prostate, 1.33 ± 3.22% for 808 head and neck (h&n), and 0.37 ± 0.62% for 108 measurements of prostate bed fields. Pelvic treatment showed differences of 0.49 ± 1.86% in 26 fields for prostate cases and 2.07 ± 2.83% in 109 fields of anal canal.

Composite measurement at isocenter for each patient showed an average difference with computed dose of 0.05 ± 0.87% for 386 prostate, 1.49 ± 1.86% for 158 h&n, 0.37 ± 0.34% for 23 prostate bed, 0.80 ± 0.28% for 4 pelvis, and 2.31 ± 0.56% for 17 anal canal cases. On the first 250 h&n analyzed by film in absolute dose, the average of the points crossing a gamma index 3% and 3 mm was 93%. This value reached 99% for the prostate fields.

**Conclusion:**

More than 3500 beams were found to be within the limits defined as validated for treatment between 2001 and 2008.

## Background

Intensity modulated radiotherapy (IMRT) was introduced in France in the early years of this century. The evolution of computing, with the ability to support new algorithms, and the implementation of multileaf collimators (MLC), made the development of this technique possible. Our Center was one of the first in France to routinely treat patients using IMRT in 2001, thus finding an efficient method of treatment delivery quality assurance (QA) was a challenge. At the beginning, no special system was developed for IMRT quality assurance so that we had to use ionization chambers and film dosimetry to perform our measurements.

Since 2001, over 1000 patients with prostate, head and neck, and anal canal carcinoma have been treated with IMRT at the Comprehensive Cancer Center of Montpellier in France. For all of them and before the first day of treatment, we have checked the dose computed by the treatment planning system (TPS) with measurements under the linear accelerator. At our institution, a single phase IMRT has been delivered for all treatments except pelvic cases [1]. Conventional treatment often required multiple portals and sequential field reductions. We hypothesized that a single phase treatment would provide the potential to reduce workload and improve radiotherapy delivery efficiency.

The number of patients who could benefit from IMRT increased dramatically as we improved our technique over the years, but conventional QA limited its widespread use because of the time needed for verification and validation of the predicted fields [2]. Indeed, clinical implementation of IMRT has been shown to be a complex process [3-8].

Every center planning to introduce this technique should be aware of the importance of such a program and allocate adequate resources to support it. Staff time has previously been shown to be greater than with conventional techniques [9].

As IMRT was becoming globally available, companies specialized in radiation measurements developed dedicated products for IMRT dosimetry, and in the last few years, electronic portal imagers have allowed the acquisition of dose produced by modulated beams and the comparison between measurement and predicted dose [10]. Further developments to reduce the burden of IMRT fields QA including validated calculation systems for the independent check of monitor units [11].

However, new technologies reducing QA time and workload should always hang in the balance with more cumbersome but reliable evidence-based methods [12]. Changing a technique that has been employed for a long time for a new one is not easily done without losing known bearings. In addition, we are now in the process of upgrading to the newer QA methods by verifying all patients plans with both techniques. A high geometric and dosimetric accuracy is required for advanced techniques, and the verification of IMRT dose distribution is a prerequisite for safe and efficient delivery [13]. At this point, there is no gold standard to set the tolerance of an IMRT plan validation, even though external audits are organized by institutions like International Atomic Energy Agency (IAEA) or European Society for Therapeutic Radiology and Oncology (ESTRO) for conventional QA [14,15] or IMRT tests plans on phantoms [16]. The question of IMRT QA is still a burning subject and as mentioned by Palta *et al*. [17]. Each facility offering IMRT have therefore to develop its own guidelines and criteria for the acceptance of IMRT QA planning and delivery systems [17]. We present here the results obtained with film and ionization chambers used during the last 8 years for dosimetry of IMRT fields.

## Methods

### Treatment planning and delivery by IMRT

Treatment plans were generated using commercial software. First studies were initially made on the Cadplan Treatment Planning System (Varian, Palo Alto, CA) in 2000, and then with Eclipse, Helios, version 7.2.34, in 2003. Three hundred and twenty segments were used to sample the sliding window delivery in the Eclipse calculation. All the plans were calculated without heterogeneity correction using a 2.5 mm dose matrix. Two linear accelerators (Varian Clinac 21 EX linear accelerator, Varian, Palo Alto, CA) were used for the treatment delivery using "sliding-window" IMRT technique with multileaf collimator (MLC Millenium 120, Varian, Palo Alto, CA). Sharing the same MLC leakage transmission, the calibration of the dosimetric leaf gap was adjusted to obtain less than 0.5% difference for the same plan delivered on the two different machines. Data were transferred from the TPS to the linear accelerator by a French record and verify system: DIC ("Dossier Informatisé en Cancérologie", Sigma Micro, Toulouse, France).

### Quality Assurance

After the treatment validation on the TPS by the physician, a QA plan was created in the system, copying all the beams included in the treatment plan on a dedicated phantom (universal IMRT phantom, PTW, Freiburg, Germany) previously scanned at our institution. All the geometry parameters could be changed but the number of monitor unit and the MLC sequence were exactly the same as for the patient plan. A specific Excel sheet was created to collect the information concerning the verification plan.

### Ionization chamber measurements

A verification plan of each field (with the gantry, table, and collimator rotations set to 0°) in the universal IMRT phantom (PTW, Freiburg, Germany) was generated in the TPS for all patients and values to specific points (holes for chambers positioning in the phantom at 6 cm depth) were considered. These points were not specially chosen in a high dose or low gradient area but were fixed by the phantom geometry. The axis dose in phantom at depth of 6 cm was measured under the accelerator using an ionization chamber with a nominal sensitive volume of 0.125 cc (PTW 31010). This detector was chosen because the configuration of the dose volume optimizer (DVO) algorithm in Eclipse was made with measurements done with this detector, even if its spatial resolution was not so small. At our department, a special interest focused on the way to configure the TPS for IMRT planning using different detectors and the influence on the fluency map created by the system. Different detectors with a smallest spatial resolution were used to commission IMRT (diamond chambers, diodes) but we finally decided to use the same detector for IMRT configuration as for the global commissioning of the linear accelerator. Other measurements for points at 2 and 4 cm lateral to the central axis could be also acquired. Nearly 500 prostate cancer patients were treated by IMRT between 2001 and 2008. All of them were treated using 6 beams (60°, 95°, 130°, 230°, 265° and 300°) and a high energy of 18 MV. For pelvic irradiation, such as anal canal or high-risk prostate cancer, split fields were used due to the large size of the target volume. Beam configuration was a 7-field template at the following gantry angles (0°, 45°, 110°, 165°, 195°, 250° and 325°). Methodology for QA was the same as for the non-split fields with measurements consisting of the sum of the different subfields for the same gantry rotation. Energy of 6 MV and 5 beams (0°, 70°, 140°, 210°, and 290°) are used with a non-split technique for the majority of the head and neck patients. For more simplicity and to spare time, the QA measurements are performed with a gantry position of 0° in a flat phantom (universal IMRT phantom, PTW, Freiburg, Germany). It is known that this method neglected the effect of gravity on the mechanical parts (gantry, MLC carriage and leaves) during our procedure. To quantify the gap that could be caused by this effect, we also irradiated the same plans in a cylindrical phantom (head and neck IMRT phantom, PTW, Freiburg) with the real gantry angle as for the treatment of the first 36 patients.

### Film dosimetry

To verify relative and/or absolute distribution in two dimensions and not simply at specific points, we decided to use film dosimetry. The film was placed for each field at 5 cm depth in the flat phantom and perpendicular to the irradiation. During the first years, we used XOMAT films (Eastman Kodak Co., Rochester, NY, USA) but the response of this film was not linear with the dose delivered. We thus replaced it with EDR2 since the latter was able to handle a dose of more than 2 Gy without any saturation effect [18]. A calibration curve was plotted each time with a verification plan for a patient. Films were developed in an automatic machine and digitized with a Vidar VXR-12 digitizer (Vidar Systems Corporation, Herndon, VA, USA)

The spatial resolution used to digitize the film was 75 dpi, which corresponded to 2.95 pixels/mm. This was not the highest resolution provided by the system but was sufficient to compare with the calculation resolution (0.338 pixels/mm compared to 2.5 pixels/mm). The information was coded in 12 bits and no filtration was used during the 10 ms of acquisition. A study was done using different digitalization tables provided by the Vidar software to see which were the most useful for routine use. We chose to look at three specific tables: linear, logarithmic, and PW5 (power 5)

**Figure 1:**
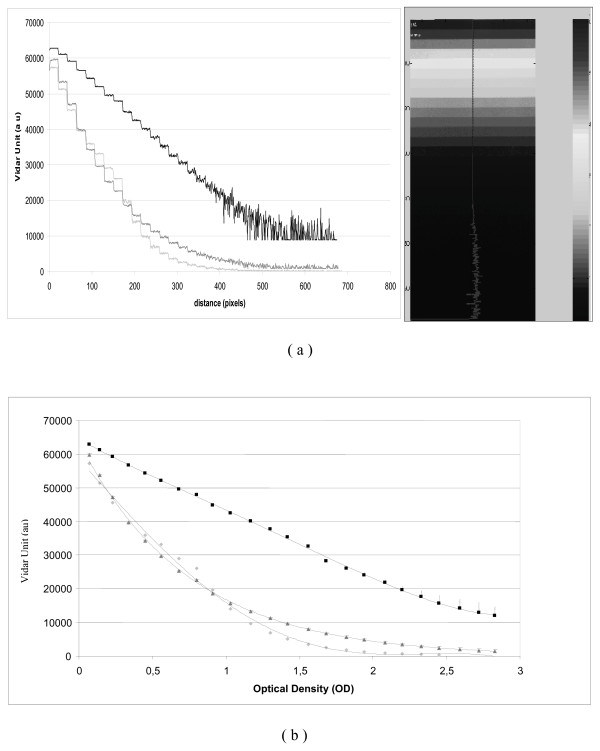
**Vidar reading (ua) of an optical density wedge**. (a) Reading of the OD step (b) Graph is plotted as a function of Optical Density for different digitalization table provide by the Vidar system. (Dark value is for logarithmic acquisition table, grey for PW5, and the light grey for linear table.)

An analysis of optical density (OD) scales provided by the Vidar VXR 12 was performed using these three different acquisition tables. A line crossing the different readings of an OD scale increasing from 0 to 3.8 OD was analyzed (Figure 1a). The electronic response of the Vidar varied with the table used (log, linear, or PW5). Logarithmic tables presented a more linear response of the signal and showed more OD levels corresponding to a higher dose. On the other hand, for low OD levels, the other tables provided a larger difference of Vidar readings for the same OD strip meaning that discrimination between two different levels was easier at smaller doses. Finally, we decided to use the log table when we verified a global plan that included the entire treatment fields.

**Figure 2:**
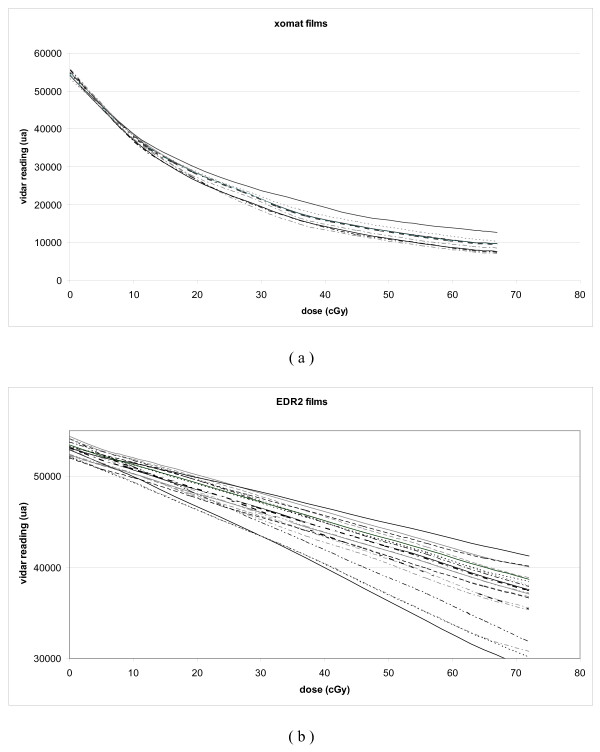
**Vidar reading (ua) for dose calibration films performed before patients QA**. XOMAT-V films (a) and EDR2 films (b).

Initially, we tried to define a calibration curve to convert the Vidar dose readings to the different acquisition table used (Figure 1b). However, we realized that, due to day-to-day variations, our film necessitated a calibration at each treatment verification. A kind of "step wedge" film with different predefined dose levels allowed us to create these calibration curves. The result for XOMAT films (Figure 2a) and EDR2 (Figure 2b) showed that it corresponded to a direct change of the slope of the curve [19].

Academic software developed by the MD Anderson hospital (Doselab) was used to analyze films by profile and isodose comparison. Since 2003, we have validated the results using the gamma index [20] but we will switch soon to radiochromic films for measurements [21].

## Results

### Ionization chamber

**Figure 3:**
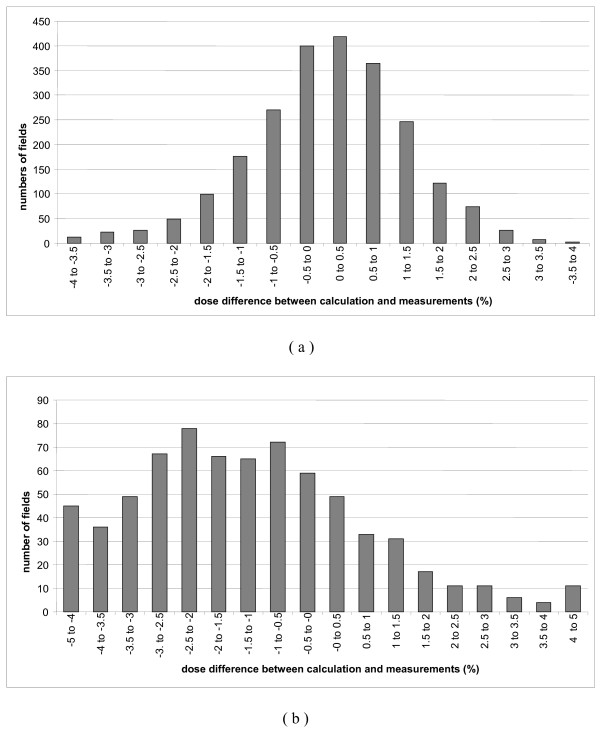
**Dose difference between measured and calculated dose for beam by beam measurements**. Results for 2319 prostate fields (a) and 808 head and neck fields (b).

We present in this paper the results for the 386 first patients corresponding to 2316 individual dose beams measured at the isocenter with the gantry at 0°. The average difference between measurements and predicted TPS dose was 0.07 ± 1.22% (Mean ± 1SD) (Figure 3a). These results are similar to a study in which 380 prostate fields were analyzed [22].

**Figure 4:**
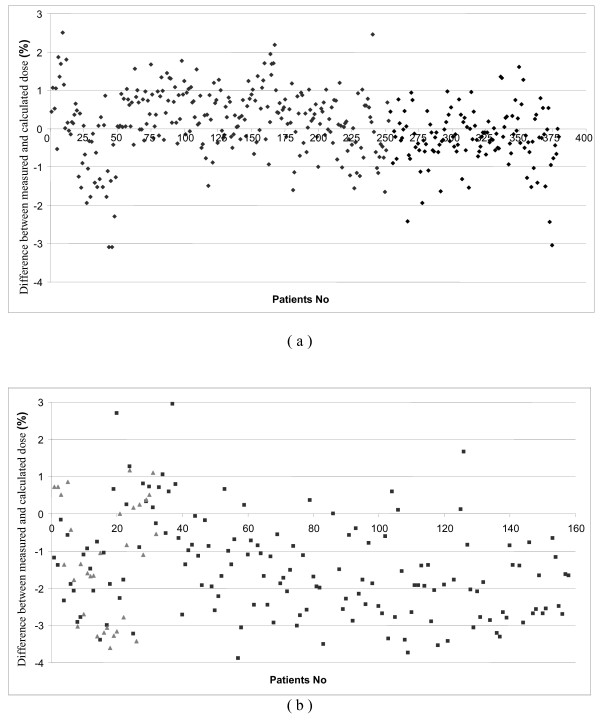
**Dose difference between measured and calculated dose for global patient verification**. Results for 383 prostate cases (a) and 158 head and neck cases (b). Square dots represent verification with gantry angle at 0° and triangular dots with the gantry in the treatment position.

Considering the dose at isocenter for the entire treatment (sum of all the beams) and for each patient, the measured dose was always within 3% of calculated dose except for 3 cases (0.05 ± 0.87%) (Figure 4a). Since 2008, IMRT has become our standard treatment for post-prostatectomy radiotherapy for which acceptable concordance was also obtained between planned and measured dose. The average difference was -0.37 ± 0.62% for the 108 beams and -0.80 ± 0.28% for the individual 23 patients.

For pelvic irradiation, the results for beam by beam analysis were -0.49 ± 1,86% and 2.07 ± 2.83% for 26 high risk prostate cancer and 109 anal canal beams, respectively. Distribution of the readings was not the same even though the energy and the beam angles were identical. Indeed, the total delivered dose was inferior for the anal canal cases compared to the high-risk prostate cancer cases (59.4 Gy vs 80 Gy ICRU) but the modulation factor was greater for anal cancer due to the increased complexity required to reach the constraints. This modulation factor could be interpreted as a new metric for assessing IMRT modulation complexity as it look at the number of MU delivered by Gy. The more difficult is the plan, the smallest is the opening of the sliding window and the number of MU necessary to deliver the dose increase. The dose distributions in prostate cases are more centered in the histogram than in anal canal cancers and similar results for the patient dose were found where discrepancies reached -0.80 ± 0.28% for 4 high-risk prostate treatments and 2.31 ± 0.56% for 17 anal canal cases.

**Figure 5:**
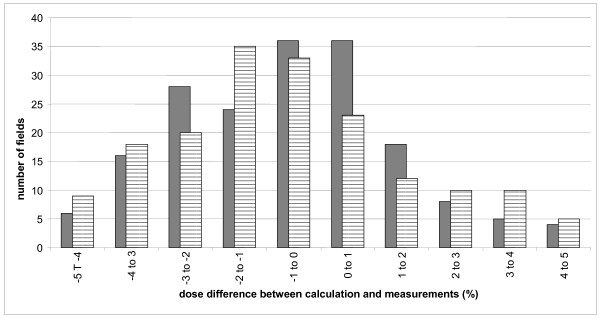
**Dose difference between measured and calculated dose for beam by beam verification for head and neck cases**. The dash bars represent acquisition realized with the gantry at the real treatment position and the full bars represent the values with the gantry at 0° for the same patients.

Head and neck treatments needed more modulation to achieve goal constraints bringing complicated fluencies that were more difficult to measure. Figure 3b shows that the shape of the graph is flatter for the 710 beam-by-beam control points of the 158 first patients (-1.33 ± 3.22%). Global measurements show more negative values than in all the other cases treated by IMRT (-1.49 ± 1.86%) (Figure 4b). The results are shown in (Figure 5) where striped bars represent measurements taken with rotated gantry. We showed that the global distribution have the same appearance with a difference between calculation and measurement of -0.70 ± 2.42% and -0.72 ± 3.20% for plan and rotated gantry studies, respectively.

### Gamma index results

As both geometric and dosimetric accuracy are important in IMRT, we decided to use the gamma (γ) index approach [23]. The dosimetric criterion is a dose-difference represented as a percentage of the prescribed dose. In the terminology of Low and Dempsey [23], the measured dose distribution was taken as the reference and the computed dose distribution was evaluated against it. If γ (i) < 1, the dose delivered at point i is considered to be within the tolerance criteria and hence is accepted with regards to the computed i.e. intended dose. It should be noted that lower γ-values are obtained by considering the full three-dimension of the calculated dose matrix and hence by incorporating dose variation in the perpendicular direction to the film, making the verification more realistic in case of longitudinal dose gradients.

**Figure 6:**
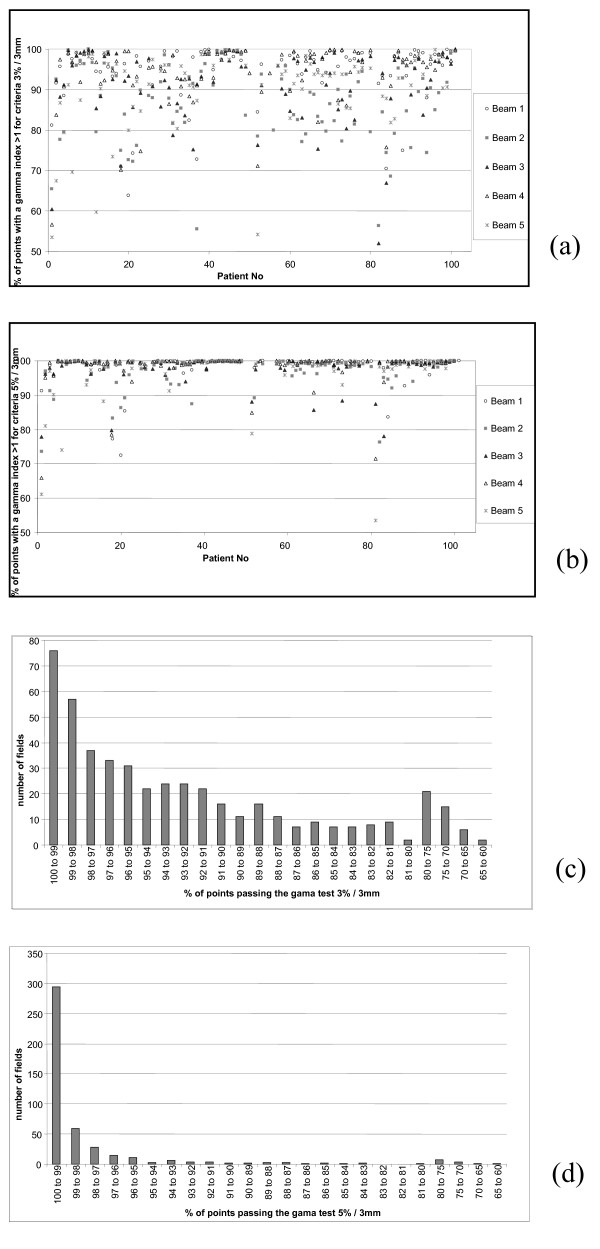
**Gama index results for the 100 first head and neck IMRT cases with dosimetric films**. Results for the 5 different beams threshold values of 3% / 3 mm (a) and 5% / 3 mm (b). Number of points that reached a gamma value < 1 for head and neck fields: Results for threshold values of 3% / 3 mm (c) and 5% / 3 mm (d).

The Doselab software was used for treatment film verification. After the verification of the calibration by applying the calibration curve to the calibration film (step wedge) and the comparison with the Eclipse plan, the calibration curve was applied to the patient films. By doing this, an absolute dose validation was possible and could be compared to IC measurements. For prostate plans, the number of points passing the gamma index was always superior to 99%. This result was in agreement with chamber measurements and was mainly due to the fact that the modulation of the beam for a prostate plan generates a uniform dose distribution in the centre of the beam which is a good condition for measurement (high dose, small gradient). Gamma histograms were calculated on the film area defined by the primary jaws. For head and neck treatments, the gamma index was studied for two different couples of values. A 3% / 3 mm criterion represented our acceptance level. We also analyzed the films with a 5% / 3 mm criterion to compare our results with those published in the literature and because the acceptance dose difference in IC for film-by-film measurements was fixed at 5%. For the 500 films studies, percentage of point reaching the gamma agreement was 91.66 ± 9.62% and 97.68 ± 5.41% for 3% / 3 mm and 5% / 3 mm, respectively (Figure 6a and Figure 6b). Some points showed a gamma index below 80% but the areas with bad results were located out of the irradiation field and inside a low (transmission only) dose area. These results are comparable to those published by De Martin et al. [24] where they showed 95.3% and 87.6% points passing the acceptance criterion of 4% / 3 mm for two therapy units and 57 head and neck patients. In their study, only points on dose levels higher than 10% of the prescription dose were studied allowing better results.

## Discussion

IMRT requires quality assurance (QA) for each patient before radiotherapy treatments but no gold standard is defined for acceptance of the verification process. Wilcox et al. [25] presented QA results on a small study of 172 patients which correlated to our measurements. The QA results are the sum of different processes in the chain of events leading to the treatment delivery. This chain can separate three different steps, each with potential errors: the treatment planning system and the optimization, the clinic and especially the multileaf collimator calibration, and the QA process.

The TPS configuration is the first subject to look at when considering IMRT QA. The way the data is initially configured and the capacity of the system to simulate real beams are crucial [26]. The sliding window technique used to deliver IMRT treatments with Varian accelerators requires configuration of Eclipse with two very important factors: the Dynamic leaf separation (DLS) and the leaf transmission (T). Many publications relate methods to determine the DLS parameters but the transmission factor is defined in a unique value [27]. The effect of this value certainly varies with the size of the primary jaws opening and with the amount of time a specific point is irradiated under the leaves [28], leading to different accuracy of measurements inside the same patient for the same energy between large fields and small fields [29]. The degree of complexity of the modulation also explains the different results for various tumor localizations. Values for the large fields used in anal canal and high risk prostates cases are completely different even if we use the same beam angles. Volume definition and protection of specific organs at risk (like iliac crests) for the first cases lead to more complicated fluencies. It means that finding a "high dose, low gradient" point to measure the dose in contrast with localized prostate cancer is difficult to reach. The standard deviation values present little importance as we wait for a standard value allowing us to rapidly determine a problem occurring in the patient preparation plan. A study of the measurement accuracy depending on the gantry angle did not show particular differences between the beams.

A specific QA procedure for the MLC is needed in case of IMRT delivery. LoSasso *et al*. [30] showed a small error in the position of the leaf during the beam delivery that could generate discrepancies between measured and calculated dose. The more complex the fluency is, the thinner the sliding window is and the more important the error generated by the poor calibration of the leaves is. The impact of this factor is more important for head and neck or anal canal cases than for localized prostate cases. External devices could be used to verify the accuracy of leaf positioning [31] or to recalibrate the MLC to obtain the DLS defined in the TPS.

The QA process itself could generate errors depending on how it is performed. Ionization chamber measurements depend on the positioning of the device and the volume collected. Chambers with a cavity bigger than 0.125 cc are not suitable for IMRT measurements. The uncertainty induced by the device itself could be estimated at 1.5% and the overall standard uncertainty of the measured IMRT dose amounts to approximately 2.3% [32]. Better results can be achieved if a "high dose/low gradient" zone is considered but, in our study, the geometry was fixed to simplify the QA process and minimize the time needed under the linear accelerator. One interest of the chamber method remains in the absolute dose collection but it is only acquired in one position of the beam compared to 2D measurements.

Because of the "poor" spatial resolution [33], arrays are the only suitable methods for verification of the reproducibility of the beam delivery. Films are the oldest method with the highest spatial resolution but are hard to use [34,35]. The software we used for the gamma index evaluation did not allow defining an analysis in the irradiated area only but in the zone defined by the primary jaws. This process gave bad results for some evaluations (worst points in Figure 6c) even if the evaluation showed good agreement inside the beam. The use of portal imager seems to be the easiest way to achieve a fast and qualitative QA for IMRT [36]. Positioning of the detector (generally attach to the Linear accelerator), spatial resolution, and dose response are more accurate with these devices. They dramatically reduce the time needed to perform the pre-treatment QA for the patient and they will allow measuring the transit dose during the irradiation. Furthermore, the measured dose is most of the time at a single point or a 2D acquisition even if 3D processes are today available [37] but remain difficult to implement in clinical routine.

QA process is still stained of uncertainty. When performing IMRT QA, physicists try to detect a systematic error in the global process of the treatment preparation without adding a random error in the QA itself. Independent calculation could probably avoid some measurement mistakes and advantageously replace measurement time under the linear accelerator [38].

In the same way, the dose delivered to the real patient, and not to a phantom, is the final goal of IMRT verification. Back calculation of the daily dose with the use of CBCT acquisition crossed a stage in the quality of the treatment follow-up [39].

## Conclusion

In this study, we report our results of more than 3500 IMRT beams control under the linear accelerator before patient treatments. Even if treatments using intensity modulation have been delivered since more than one decade, a lot of centers in the world are starting this technology. The goal of this paper is to provide an important number of measurements and to develop the understanding of the results quality depending on the implemented assurance process. Our results show that sliding window technique is robust and can be applied to various tumor sites. A localization effect appeared as we introduced new patients to IMRT, but differences between measurements and calculated dose remained 5%. Conventional methods using ionization chambers and film dosimetry are used and are still robust but new technologies are now available giving equivalent results together with decreased time needed in the treatment room. Since 2008, we have replaced our technique to EPID for all IMRT measurements but we still use our old QA method to validate software upgrade.

## Competing interests

The authors declare that they have no competing interests.

## Authors' contributions

PF, BL conceived the study, collected data, and drafted the manuscript. NA, JBD,OR and DA participated in coordination and helped to draft the manuscript. DA provided mentorship and edited the manuscript. All authors have read and approved the final manuscript.
